# Fluid resuscitation via colon alleviates systemic inflammation in rats with early-stage severe acute pancreatitis

**DOI:** 10.1038/s41598-021-96394-5

**Published:** 2021-08-19

**Authors:** Tongtian Ni, Lili Xu, Silei Sun, Li Ma, Bing Zhao, Weijun Zhou, Yi Wen, Ning Ning, Erzhen Chen, Ying Chen, Enqiang Mao

**Affiliations:** Department of Emergency, Ruijin Hospital, Affiliated To Shanghai Jiao Tong University School of Medicine, No. 197, Ruijin er Road, Huangpu District, Shanghai, 200025 China

**Keywords:** Pancreatitis, Acute inflammation

## Abstract

Fluid resuscitation via colon (FRVC) is a complementary therapeutic procedure for early-stage cases of severe acute pancreatitis (SAP). The expression of intestinal dendritic cell-specific intercellular adhesion molecule 3-grabbing nonintegrin (DC-SIGN) regulates systemic inflammation. This study aimed to investigate the effect of FRVC on the expression of DC-SIGN in the colon tissue of SAP rats and its effect on the early response of systemic inflammatory and multiple organ injury. SAP was induced in rats via retrograde injection of sodium taurocholate into the biliopancreatic duct. DC-SIGN expression of appeared in the proximal and distal colon. Histological characteristics and inflammatory cytokines were examined to compare the effect of FRVC and intravenous fluid resuscitation (IVFR). The results showed that DC-SIGN expression in the proximal colon increased in a time-dependent manner in the early-stage of SAP rats. FRVC inhibited DC-SIGN expression in the proximal colon. Both FRVC and IVFR alleviated histological injuries of the pancreas and colon. However, FRVC had an advantage over IVFR in alleviating lung injury and reducing serum TNF-α, IL-6 and LPS. These results suggest that FRVC treatment might help suppress systemic inflammation and prevent subsequent organ failure in early-stage SAP rats likely through inhibiting DC-SIGN expression in the proximal colon.

## Introduction

Severe acute pancreatitis (SAP) is a severe systemic disease characterized by acute inflammation and necrosis of the pancreas and peripancreatic tissues^1^. It is a subtype of acute pancreatitis accompanied by multiple organ dysfunction syndrome (MODS) and systemic inflammatory response syndrome (SIRS)^2^. Although its mortality rate is gradually decreasing as treatment modalities improve^3^, it remains a lethal disease with a mortality rate between 20 and 40%^1^. Fluid resuscitation to prevent hypovolemia and organ hypoperfusion is the main treatment of SAP. Although the classic intravenous fluid resuscitation (IVFR) can significantly reduce the mortality of patients with SAP^4,5^, the body passively receives fluid infusion in this state. Howerer, the absorption of fluid by the colon is an active process. Fluid resuscitation via colon (FRVC) can significantly improve hemodynamics, and reduce pathological lung and liver damage in rats with SAP^6^. However, until now the mechanism remains unclear.

Dendritic cell-specific ICAM-3-grabbing nonintegrin (DC-SIGN) is a DC-specific C-type lectin-like cell-surface receptor that binds intercellular adhesion molecule-3 (ICAM-3) and intercellular adhesion molecule-2 (ICAM-2) on T cells, promoting the adhesion of DCs to naive T cells^7^. It plays an important role in the intestinal immune response^8^. Intestinal epithelial cells undergo epithelial-dendritic cell transformation by expressing DC-SIGN^9^. A previous study has shown that DC-SIGN inhibition could reduce the intestinal and systemic inflammatory responses and the mortality in septic mice^10^.

In the view of this background, we hypothesized that FRVC could regulate the early inflammatory response in SAP rats by affecting DC-SIGN expression in the colon. This study aimed to investigate the effect of FRVC on the expression of DC-SIGN in the colon tissue of SAP rats and its effect on the early systemic response inflammatory and multiple organ injury.

## Results

### DC-SIGN expression in the proximal colon of SAP rats increased time-dependently

**Figure 1:**
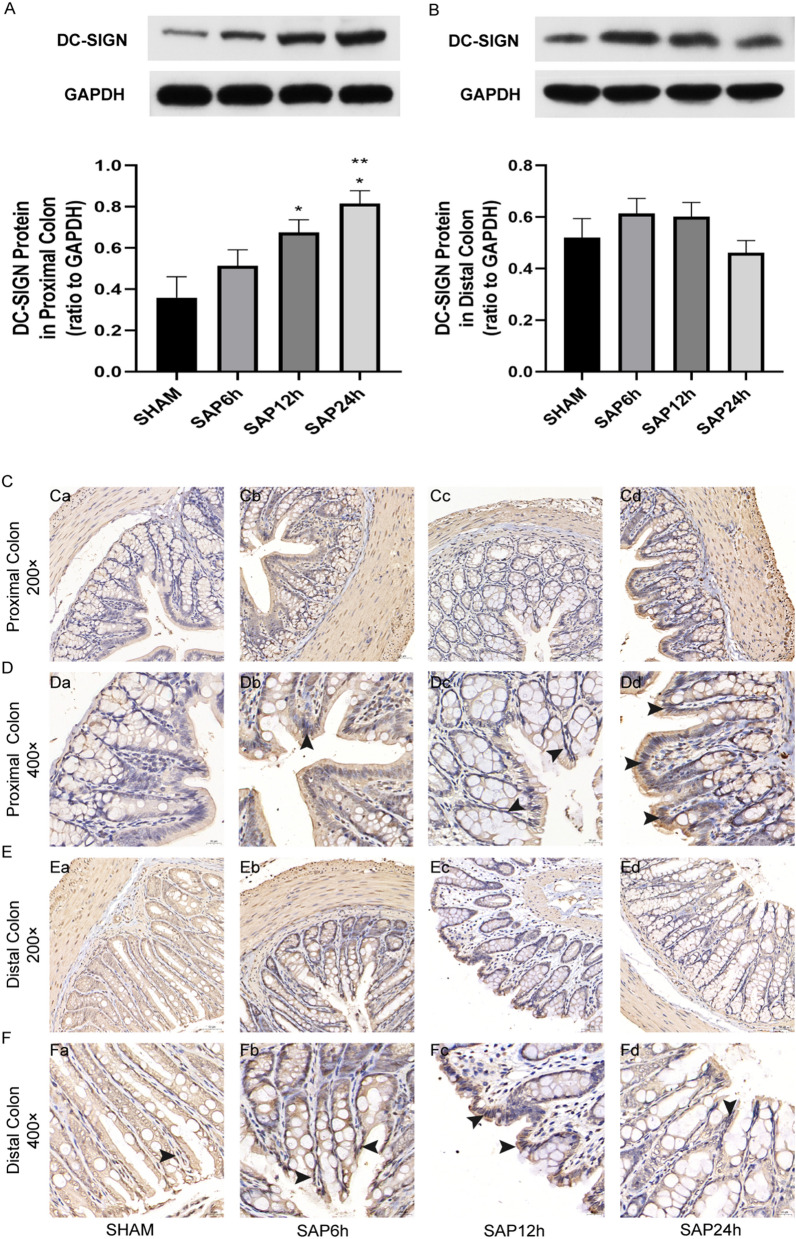
DC-SIGN expression in the proximal and distal colons of SAP rats at 6, 12, and 24 h. (**A**) Western blot analysis of DC-SIGN expression in the proximal colon. (**B**) Western blot analysis of DC-SIGN expression in the distal colon. (**C**,**D**) Immunohistochemical staining of DC-SIGN expression in the proximal colon. (**E**,**F**) Immunohistochemical staining of DC-SIGN expression in the distal colon. *p < 0.05 compared to SHAM, **p < 0.05 compared to SAP6h.

The protein expression of DC-SIGN was detected both in the proximal and distal colon of SAP rats. DC-SIGN expression was almost undetectable in the proximal colon tissues of the sham group. It increased significantly in the 12 h and 24 h SAP models (*P* < 0.05) (Fig. 1A), while the expression of DC-SIGN in the distal colon tissues of the sham group was very low and time-independent (Fig. 1B). The immunohistochemical staining demonstrated that DC-SIGN expression mostly occurred in the colonic epithelial cells of SAP rats (Fig. 1C–F).

### FRVC inhibits DC-SIGN expression in the proximal colon of early-stage SAP rats

**Figure 2:**
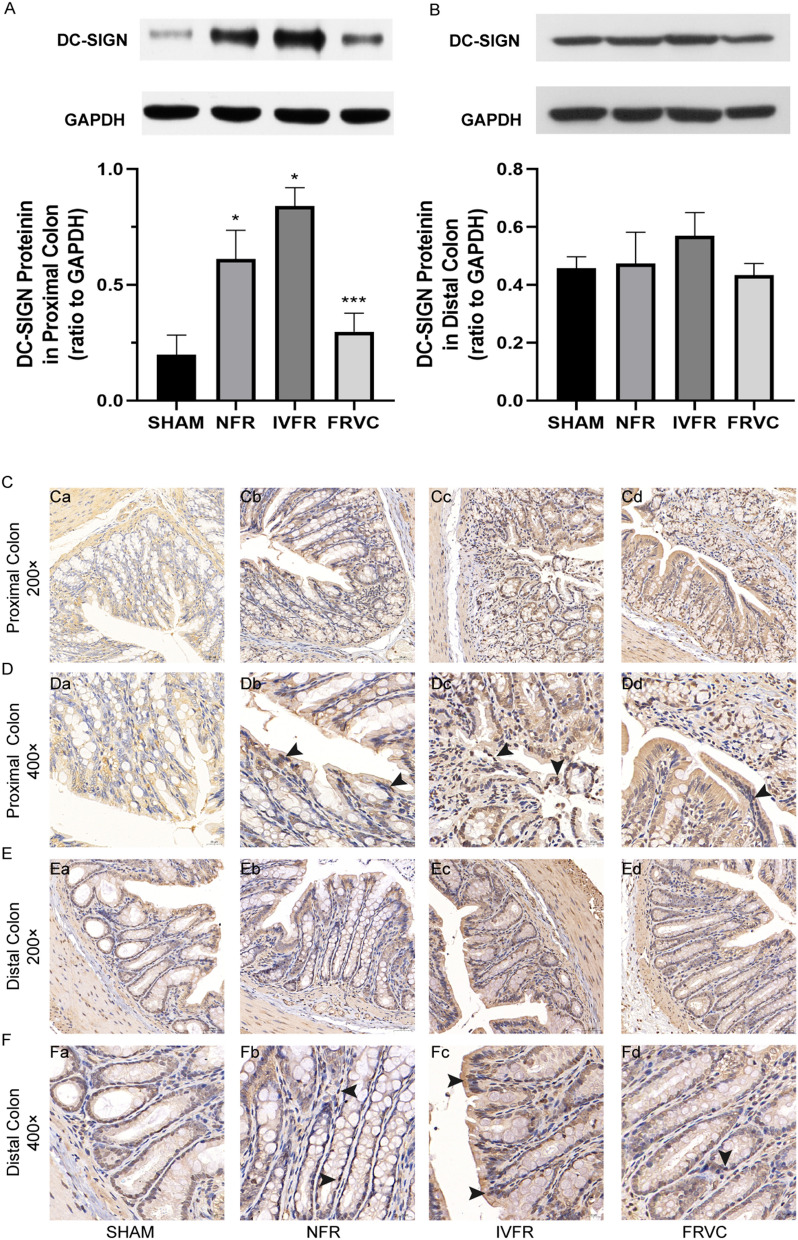
FRVC inhibits the expression of DC-SIGN in the proximal colon of SAP rats. (**A**) Western blot analysis of DC-SIGN expression in the proximal colon. (**B**) Western blot analysis of DC-SIGN expression in the distal colon. (**C**,**D**) Immunohistochemical staining of DC-SIGN expression in the proximal colon. (**E**,**F**) Immunohistochemical staining of DC-SIGN expression in the distal colon. *p < 0.05 compared to SHAM, **p < 0.05 compared to NFR; ***p < 0.05 compared to IVFR. SHAM: the rats treated with sham operation and no fluid resuscitation; NFR: the rats treated with the SAP operation and no fluid resuscitation; IVFR: the rats treated with the SAP operation and intravenous normal saline infusion; FRVC: the rats treated with the SAP operation and normal saline infusion via colon.

Western blot analysis revealed that DC-SIGN expression in the proximal colon increased in the IVFR group compared with the non-fluid resuscitation (NFR) group, but this was statistically significant. FRVC treatment significantly inhibited DC-SIGN expression in the proximal colon compared with the IVFR treatment (*P* < 0.05) (Fig. 2A). A similar trend occurred in the distal colon, but there was no significant statistical difference (Fig. 2B). Immunohistochemistry also revealed that DC-SIGN expression in the proximal colon epithelium cells was markedly increased after IVFR treatment but reduced after FRVC treatment (Fig. 2C,D); changes in the distal colon were not obvious (Fig. 2E,F).

### FRVC alleviates organ damage in early-stage SAP rats

**Figure 3:**
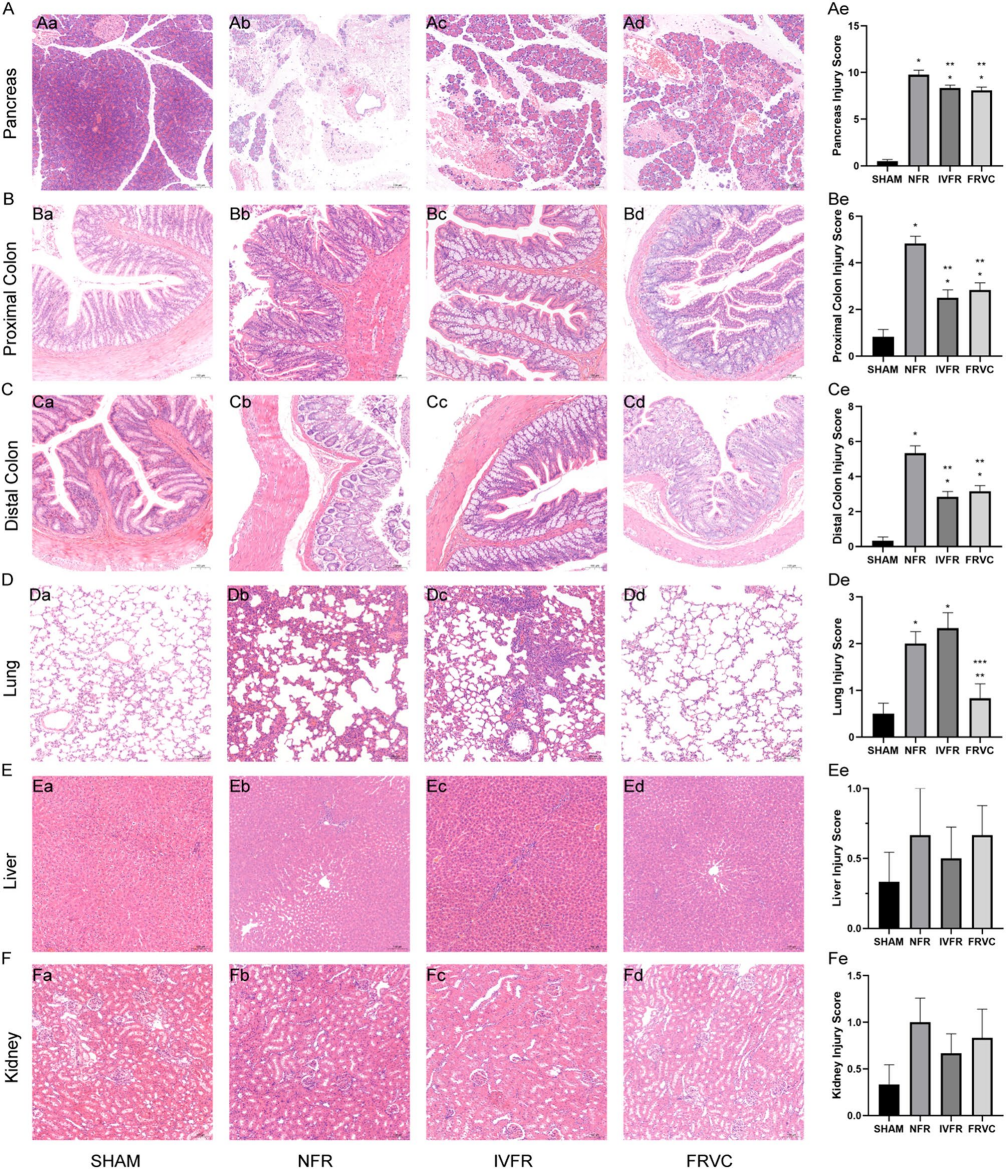
Organ damage of SAP rats and SAP rats treated with IVFR or FRVC. (**A**–**F**) The severity of pancreas, proximal colon, distal colon, lung, liver, and kidney injury was assessed via H&E staining and histological scores. Magnification: 100×. Scale bar: 100 μm. *p < 0.05 compared to SHAM, **p < 0.05 compared to NFR; ***p < 0.05 compared to IVFR. SHAM: the rats treated with sham operation and no fluid resuscitation; NFR: the rats treated with the SAP operation and no fluid resuscitation; IVFR: the rats treated with the SAP operation and intravenous normal saline infusion; FRVC: the rats treated with the SAP operation and normal saline infusion via colon.

On hematoxylin and eosin (H&E) staining, histological damage of the pancreas was significantly worse in the NFR group compared with the SHAM group 24 h after the induction of SAP (*P* < 0.05) (Fig. 3A). However, after both IVFR and FRVC treatment, the histological damage of the pancreas was significantly relieved (*P* < 0.05). But there was no significant difference between the two treatment groups. Similarly, the proximal and distal colon histological damage was significantly different between the SHAM group and the NFR group (*P* < 0.05) (Fig. 3B,C), both improved after treatment with IVFR and FRVC (*P* < 0.05). Obvious histological damage was observed in the lung on H&E staining (*P* < 0.05) (Fig. 3D), but IVFR did not reduce the damage. In contrast, FRVC treatment significantly improved lung injury than NFR and IVFR (*P* < 0.05). The liver and kidney histological damage were mild, with no significant difference between the four groups (Fig. 3E,F).

### FRVC reduced systemic cytokines levels of SAP rats in early-stage

**Figure 4:**
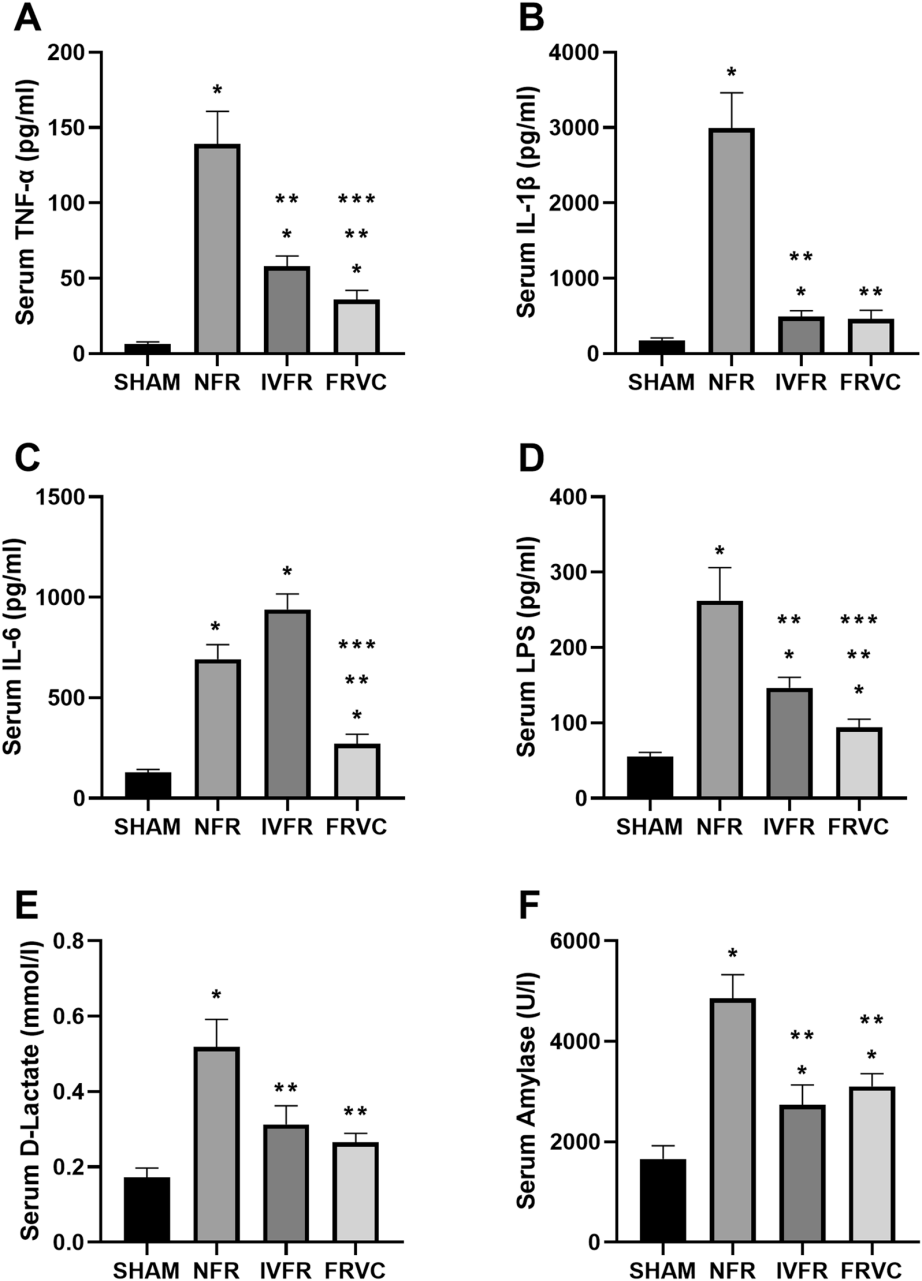
Changes in levels of systemic cytokines in SAP rats and SAP rats treated with IVFR or FRVC. (**A**–**F**) Serum levels of TNF-α, IL-1β, IL-6, LPS, D-lactate, and amylase were analyzed via ELISA. *p < 0.05 compared to SHAM, **p < 0.05 compared to NFR; ***p < 0.05 compared to IVFR. SHAM: the rats treated with sham operation and no fluid resuscitation; NFR: the rats treated with the SAP operation and no fluid resuscitation; IVFR: the rats treated with the SAP operation and intravenous normal saline infusion; FRVC: the rats treated with the SAP operation and normal saline infusion via colon. TNF-α, tumor necrosis factor-α; IL-1β, interleukin-1β; IL-6, interleukin-6; LPS, lipopolysaccharide.

The serum levels of TNF-α, IL-1β, IL-6, LPS, D-lactate, and amylase were greatly increased in the NFR group compared to the sham group (*P* < 0.05) (Fig. 4). After IVFR, the serum levels of TNF-α, IL-1β, LPS, D-lactate and amylase of SAP rats significantly decreased (*P* < 0.05). All six serum indicators were also significantly decreased compared between the NFR and FRVC groups (*P* < 0.05). Moreover, the FRVC group also had significantly decreased serum levels of TNF-α, IL-6, and LPS compared to the IVFR group (*P* < 0.05).

## Discussion

To our knowledge, this is the first study to investigate how FRVC reduces the inflammatory response in early-stage SAP rats. DC-SIGN expression in the proximal colon of SAP rats was found to be time-dependent. In addition, FRVC could down-regulate DC-SIGN expression in the proximal colon of SAP rats and reduce inflammation to attenuate lung injury.

In the present study, increased DC-SIGN expression was observed in the proximal colon of SAP rats. This expression was time-dependent within 24 h after induction of SAP. Intestinal epithelial cells play an antigen presentation function after DC-SIGN expression to regulate the immune inflammatory response^11^. Under chronic inflammation, DC-SIGN expression in epithelial cells of the stomach, intestine and renal tubules promotes the differentiation of CD4 + T cells to Th1 or Th2 cells and the secretion of cytokines^12,13^. A recent study^10^ has reported that DC-SIGN expression in intestinal epithelial cells can also be up-regulated in septic mice. The mechanism of differential expression of DC-SIGN in the proximal and distal colon can be attributed to the difference in abundance of various bacteria in the proximal and distal colon of rats^14^. During critical illness, a large amount of fucosidase released by specific intestinal bacteria can hydrolyze glycosylated proteins to release a large amount of fucose^15^. Fucose is also the classic ligand of DC-SIGN, which can activate and regulate the expression of DC-SIGN^16^.

In our study, DC-SIGN expression in the proximal colon of SAP rats was mainly observed in colonic epithelial cells and could be inhibited by treatment with FRVC. However, it is unclear whether DC-SIGN originates from the migration of immature DC from the circulatory system to the inflammatory tissue. DC-SIGN is also expressed in macrophages^17^, which are involved in the development of pancreatitis; modulating macrophage function in turn helps modulate the severity of pancreatitis^18,19^. Our research was not able to determine the precise source of DC-SIGN expression. However, using siRNA to inhibit the expression of DC-SIGN in intestinal epithelial cells can significantly inhibit the intestinal immune inflammatory response^10^. FRVC, a procedure that evolved from the enema, involves injecting liquid directly and continuously into the colon. When performing enema for early-stage SAP rats, it was accidentally found that the colon could quickly absorb a large amount of fluid^6^. This phenomenon was believed to reduce the frequency of infectious complications by reducing bacterial translocation^20^ and allowing the active ingredients of certain drugs to be absorbed into the blood circulation^21,22^. Therefore, we speculate that while FRVC inhibits the expression of DC-SIGN in the proximal colon of SAP rats, it may also inhibit the intestinal immune response.

In our study, IVFR or FRVC can improve histological damage of the pancreas, proximal colon, and distal colon. The histopathological scores of the pancreas improved in the IVFR and FRVC groups were improved compared with the NFR group, with no significant difference between the IVFR and FRVC groups. This might be caused by the failure of fluid resuscitation to relieve pancreatic duct obstruction, although fluid resuscitation could reduce damage to the pancreas. The link between bowel injury, gut barrier damage, bacterial translocation, and organ failure is very close^23^. Several reports^24–26^ have shown that intestinal epithelial cells have unique immune regulation functions and participated in the intestinal immune inflammatory response. Many inflammatory factors produced by uncontrolled intestinal immune inflammatory responses enter the blood circulation through the lymphatic pathway, leading to SIRS and MODS. Intravenous infusion of mesenteric lymph from severe intraperitoneal infection rats induce lung injury in healthy rats^27^, while mesenteric lymph duct ligation attenuates lung injury after intraperitoneal injection of endotoxin in rats^28^. All these suggest that inhibiting the uncontrolled intestinal immune inflammatory response is an effective measure to block SIRS and MODS. Only histological injuries of the lung demonstrated a significant improvement in the FRVC group. This can be attributed to FRVC reducing serum inflammatory mediators in SAP rats. In our study, the degree of liver and kidney damage was mild and not always observed, and there was no statistical difference in liver and kidney injury scores between the IVFR and FRVC groups.

To confirm successful induction of acute pancreatitis, we monitored serum amylase to adhere to international agreements on the experimental models of pancreatitis^29^. The present study also demonstrated that FRVC reduced systemic inflammatory factors in the serum at the same time. Although IVFR and FRVC helped reduce serum TNF-α, IL-1β, LPS and D-lactate, FRVC reduces serum TNF-α and LPS to a greater extent. LPS interacts with Toll-like receptor 4 (TLR4) to initiate a complex signal pathway and results in a pro-inflammatory response that damages the lungs^30^. This further explains the cause of lung histological damage. Both TNF-α and IL-6 mediate injury in acute pancreatitis^31^. Thus, IL-6 level is an important indicator for predicting SAP^32^, and this decreased significantly in the FRVC group. Our results also showed that D-lactate increased in the NFR group, and decreased significantly after fluid resuscitation. D-lactate level can be used to assess intestinal barrier dysfunction^33^. The intestinal permeability of SAP rats increases, resulting in a significant increase in D-lactate^34^. These findings suggest that FRVC has a greater effect on reducing the early systemic inflammatory response in SAP rats.

There are some limitations in this study. First, since establishing the SAP rat model of FRVC requires continuous anesthesia for up to 12 h, the rats in the SHAM group were also continuously anesthetized for 12 h to eliminate the influence of anesthesia. This might have affected the pathophysiology of the rat and hemodynamic parameters were not evaluated. Second, during pancreatic duct perfusion, the intestines may become dry when exposed to the air, affecting intestinal DC-SIGN expression. Third, it is very difficult to calculate how much the colon absorbs normal saline. When the catheter used for fluid resuscitation was removed, some normal saline was discharged from the intestine. Furthermore, normal saline could have been excreted when the rats were awake. Lastly, the present study did not further explore the relationship between DC-SIGN expression in the colon of SAP rats and lung injury. Further research is needed to investigate the effect of colon DC-SIGN expression on lung injury.

## Conclusion

In summary, this is the first study to show the mechanism of FRVC in the treatment of SIRS and MODS in early-stage SAP rats. We demonstrated that, in early-stage SAP rats, inhibiting DC-SIGN expression in the proximal colon via FRVC reduced the histological damage of the pancreas, colon, and lungs; the levels of systemic inflammatory factors are also reduced. These results indicate that FRVC, as a supplementary method for intravenous fluid resuscitation, might help suppress early-stage SIRS and prevent subsequent organ failure. Nevertheless, the relationship between DC-SIGN and organ damage still needs further study.

## Materials and methods

### Animals

Clean grade healthy Sprague–Dawley (SD) rats (male, 6–7 weeks, 250–300 g) were purchased from Zhejiang Vital River Laboratory Animal Technology Company Limited. All rats were housed in a controlled, air-conditioned environment (ambient temperature: 25 ± 0.5 °C, humidity: 50–60%) with a 12/12-h light/dark cycle in the animal experiment center of Ruijin Hospital. Food and water were freely accessible by rats. The study was performed in accordance with the Principles of Laboratory Animal Care (NIH publication no.85Y23, revised 1996) and in compliance with the ARRIVE guidelines. All experiments were approved by the Animal Ethics Committee of Ruijin Hospital, affiliated to Shanghai Jiao Tong University School of Medicine, and carried out according to the institutional guidelines.

### SAP modeling and experimental design

In the first stage, twenty-four rats were randomly assigned into four groups (n = 6 in each group) to observe the expression of DC-DIGN in colon tissue: sham group, SAP 6 h group, SAP 12 h group and SAP 24 h group. After screening out the time point with the highest expression of DC-SIGN in colon tissues, 24 more rats were divided into four groups according to the random number table, namely sham group, non-fluid resuscitation (NFR) group, IVFR group and FRVC group.

The SAP model was established according to the method described by Aho et al.^35^. The rats were anesthetized by using isoflurane (RWD Life Science, Shenzhen, China). The pancreatic duct at the tail of the pancreas was blocked using a vascular clip, then a closed venous indwelling needle (BD Company, Shanghai, China) was retrogradely penetrated into the pancreatic duct. 5% sodium taurocholate solution (0.1 mL/100 g body weight, 3 mL/h, Sigma, United States) was injected into the pancreatic duct with a microinjection pump. Before pulling out the needle, pressure in the pancreatic duct was maintained for 5 min.

IVFR and FRVC were performed in SAP according to the previous study^6^ after the SAP models were completed. For IVFR, normal saline was continuously infused at a rate of 4 ml/kg/h for 12 h using a microinjection pump through a Y-type trocar implanted in the right femoral vein. For FRVC, a Swan-Ganz floating catheter was inserted about 4 cm from the anus, and about 0.5 ml of air was injected into the balloon to fix the catheter. The end of the catheter was connected to a microinjection. The liquid was also infused into the colon at a rate of 4 ml/kg/h for 12 h. In the sham group, the abdominal cavity of rat was opened and closed without performing other operations. The serum samples and tissues were immediately isolated and stored at − 80 °C until analysis.

### Western blot

Protein concentration was measured using a BCA protein assay kit (Servicebio, Wuhan, China). Specifically, 20 μg of the protein samples were loaded onto 10% sodium dodecyl sulfate–polyacrylamide gel for electrophoresis and transferred to polyvinylidene fluoride membranes (Millipore, Temecula, Calif). After being blocked with 5% skim milk at room temperature for 1 h, the membranes were incubated overnight at 4 °C with primary rabbit antibodies of DC-SIGN (1:2000, Thermo Fisher Scientific, Shanghai, China) and with HRP-conjugated secondary antibodies for 1 h at room temperature. The blots were detected via chemiluminescence using the ECL reagent (Servicebio, Wuhan, China). The results were visualized using darkroom development techniques. The bands were analyzed with the AlphaEaseFC software and compared with GAPDH.

### Immunohistochemical

For the immunohistochemical detection of DC-SIGN expression, tissue sections on glass slides were placed in the citrate antigen retrieval solution (pH 6.0) to retrieve the antigen. After being treated with endogenous peroxidase and nonspecific protein blocking, the sections were overnight incubated with DC-SIGN primary antibody (1:100, A01025-2, Boster Biological Technology Co., Ltd., Calif) at 4℃ and then washed three times for 5 min each with PBS. Afterward, the sections were incubated with biotinylated secondary antibody (1:200, GB23303, Servicebio, Wuhan, China) for 1 h at room temperature. Lastly, these were stained with diaminobenzidine for microscopic examination.

### Histological analysis

The pancreas, colon, lung, liver, and kidney tissues were fixed in 4% paraformaldehyde for 24 h, then dehydrated and waxed. Afterward, these were embedded and cut into 4-μm thick slices for H&E staining. Two senior pathologists individually and blindly scored the tissues using an optical microscope. All histopathologic evaluations were performed on six fields per section under 100 × magnification and scored according to the previous study^36–38^.

### Enzyme-linked immunosorbent assays

Enzyme-linked immunosorbent assays (ELISAs) were performed with the serum samples of rats using commercial rat-specific kits for tumor necrosis factor-α (TNF-α), interleukin-1β (IL-1β), interleukin-6 (IL-6), lipopolysaccharide (LPS) (Multi Sciences Biotech Co., Ltd. Hangzhou, China), D-lactate (Abcam, Cambridge, UK), and amylase (Jiancheng Bioengineering Institute, Nanjing, China) according to the product specifications.

### Statistical analysis

The clinical data was analyzed using the SPSS 19.0 statistical software (SPSS, Inc., Chicago, IL) and GraphPad Prism 6 (GraphPad Software, San Diego, Calif). Differences between groups were analyzed using a one-way ANOVA and Mann–Whitney test. Data are expressed as mean ± standard error of mean (SEM). *P* < 0.05 was considered statistically significant.

## Supplementary Information

Supplementary Figure S1.

## Data Availability

The data used to support the findings of the study is available from the corresponding author upon request.
