# Supplementary material for: Fluid resuscitation via colon alleviates systemic inflammation in rats with early-stage severe acute pancreatitis
Source: Sci Rep. 2021 Aug 19;11:16836. doi: 10.1038/s41598-021-96394-5 (PMC8376880; doi:10.1038/s41598-021-96394-5)

Fluid Resuscitation Via Colon alleviates systemic inflammation in early stage of rats with severe acute pancreatitis

Tongtian Ni[#], Lili Xu[#], Silei Sun, Li Ma, Bing Zhao, Weijun Zhou, Yi Wen, Ning Ning, Erzhen Chen, Ying Chen*, Enqiang Mao*

Department of Emergency, Ruijin Hospital, affiliated to Shanghai Jiao Tong University School of Medicine, Shanghai, China

Supplementary Figure S1. Uncropped western blot images corresponding to Figure 1A(a), Figure 1B(b) ,Figure 2A(c). Figure 2B(d)

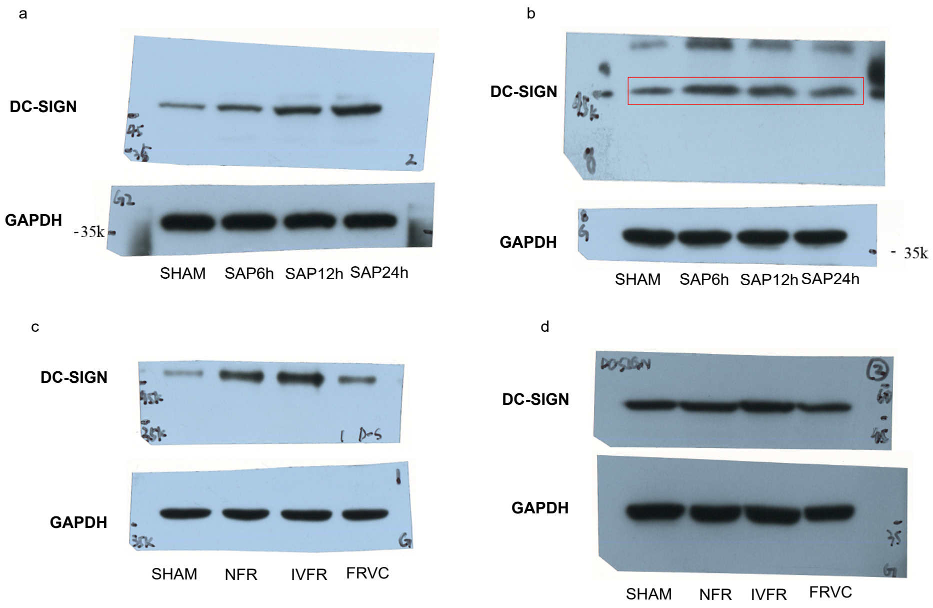

Supplement: Supplementary file 1 — Supplementary Figure S1. [file 41598_2021_96394_MOESM1_ESM.pdf]
